# Efficacy of MAVIG X-Ray Protective Drapes in Reducing CTO Operator Radiation

**DOI:** 10.1155/2021/3146104

**Published:** 2021-12-14

**Authors:** Keir McCutcheon, Maarten Vanhaverbeke, Jérémie Dabin, Ruben Pauwels, Werner Schoonjans, Walter Desmet, Johan Bennett

**Affiliations:** ^1^Department of Cardiovascular Medicine, University Hospitals Leuven, Leuven, Belgium; ^2^Department of Cardiovascular Sciences, Katholieke Universiteit, Leuven, Belgium; ^3^Belgian Nuclear Research Centre, Research in Dosimetric Applications, Mol, Belgium

## Abstract

**Background:**

The MAVIG X-ray protective drape (MXPD) has been shown to reduce operator radiation dose during percutaneous coronary interventions (PCI). Whether MXPDs are also effective in reducing operator radiation during chronic total occlusion (CTO) PCI, often with dual access, is unknown.

**Methods:**

We performed a prospective, randomized-controlled study comparing operator radiation dose during CTO PCI (*n* = 60) with or without pelvic MXPDs. The primary outcomes were the difference in first operator radiation dose (*μ*Sv) and relative dose of the first operator (radiation dose normalized for dose area product) at the level of the chest in the two groups. The effectiveness of MXPD in CTO PCI was compared with non-CTO PCI using a patient-level pooled analysis with a previously published non-CTO PCI randomized study.

**Results:**

The use of the MXPD was associated with a 37% reduction in operator dose (weighted median dose 26.0 (IQR 10.00–29.47) *μ*Sv in the drape group versus 41.8 (IQR 30.82–60.59) *μ*Sv in the no drape group; *P* < 0.001) and a 60% reduction in relative operator dose (median dose 3.5 (IQR 2.5–5.4) E/DAPx10^−3^ in the drape group versus 8.6 (IQR 4.2–12.5) E/DAPx10^−3^ in the no drape group; *P*=0.001). MXPD was equally effective in reducing operator dose in CTO PCI compared with non-CTO PCI (*P* value for interaction 0.963).

**Conclusions:**

The pelvic MAVIG X-ray protective drape significantly reduced CTO operator radiation dose. This trial is clinically registered with https://www.clinicaltrials.gov (unique identifier: NCT04285944).

## 1. Introduction

The main source of operator radiation during percutaneous coronary interventions (PCI) is scatter from the patient. Reduction of this radiation is especially important in chronic total occlusion (CTO) PCI since these procedures are lengthy and are mostly carried out by dedicated operators. While CTO operator radiation can be reduced by adjustment of catheterization suite settings, there is potential for further improvement [1]. Several studies have demonstrated that operator radiation during routine cardiac catheterization procedures can be substantially reduced by pelvic drapes [2, 3]. MAVIG X-ray protective drape® (MAVIG, Munich, Germany) are widely available, relatively small, light-weight, and reusable. These drapes have been designed to protect against radiation emitting from the patient's body during both femoral and radial access procedures, and we have previously demonstrated that they reduce operator radiation dose by half during routine cardiac catheterization and PCI [4]. However, there is very little data on the effect of these devices during CTO PCI, which often involves dual access. Here, we present the results of a prespecified parallel prospective randomized-controlled trial (RCT) where we measured operator radiation dose during CTO procedures with and without MAVIG X-ray protective drapes.

## 2. Methods

Consecutive CTO procedures were included from October 2019 until November 2020 as part of a single-center, investigator-initiated RCT (ClinicalTrials.gov Identifier NCT04285944). During study design, it was anticipated that inclusion of CTO procedures would take much longer than inclusion for routine cardiac catheterization procedures. Therefore, a separate parallel study, with separate randomization and separate dosimeters, was planned. All drapes had already been purchased by the University Hospitals Leuven, and no funding was obtained from MAVIG (Munich, Germany). The study protocol was approved by the local ethical committee (study identifier S62469). Details of the methods, the cardiac catheterization facility, radiation measurements, and statistical analysis have been published previously [4]. The details of the catheterization theatres have been previously published, and no interaction between the treatment effect and theatre was found (*P* value for interaction 0.113).

The MXPDs have been designed to protect against scatter radiation from the patient's body during both femoral and radial access procedures. The femoral drape (ST-FS5AMM) weighs 1.31 kg and measures 75 × 36 cm. The radial drape (ST-RZ5AMM) weighs 1.56 kg and measures 75 × 40 cm. These drapes have a lead equivalence of 0.5 mm. The MXPDs were inserted into a sterile sleeve and positioned over the pelvic area of the patient with the larger portion of the MXPD placed towards the operator.

We used a computer-based 1 : 1 randomization, which took place prior to the start of the cardiac catheterization procedure. Depending on the result, the first and second operators (who stood in the first operator and second operator positions on the right of the table, near the pelvis of the patient) wore the dosimeters for the MXPD arm or the dosimeters for the control arm. Study dosimeters were shared by CTO operators and analysed after ten procedures in order to increase the accuracy of the operator radiation dose measurement. For every procedure, the operators wore chest dosimeters (Inlight, Landauer, USA) on the outside of the left apron pocket. Dosimeters were calibrated according to ISO norms N60 reference beam against personal dose equivalent Hp(10), Hp(3), and Hp(0.07), respectively [5, 6]. The uncertainty (*k* = 1) associated with the measurements was conservatively estimated to be 40% for doses smaller than 40 *μ*Sv and 10% otherwise. The dosimeter readings and analysis were performed at a separate facility (Belgian Nuclear Research Centre, Research in Dosimetric Applications, Mol, Belgium).

The primary endpoints were the difference in operator radiation dose between the drape and no drape groups, and the difference in operator radiation dose indexed for the patient radiation dose as estimated by the dose area product (E/DAP). CTO operator dose was a summation of first and second operator radiation dosimeters, since the operators switched position in 27% of cases.

Continuous variables are expressed as mean ± standard deviation (SD) or median and interquartile range (IQR, 25^th^–75^th^ quartile) depending on the distribution of the data. Categorical data are reported as *n* (%). The average dose (E, *μ*Sv) per procedure was calculated by dividing the total exposure by the number of procedures recorded on the dosimeter. Radiation dose is reported as the weighted median and interquartile range, in which the weighting factor accounts for the number of procedures recorded per dosimeter. The weighted difference in dose per procedure between the two groups was assessed using the Mann–Whitney *U* test. Statistical analyses were performed using SPSS Version 24.0 (IBM Corp., N.Y., U.S.A.), applying a significance level of ≤0.05. The data will be made available to other researchers upon reasonable request.

## 3. Results

**Figure 1:**
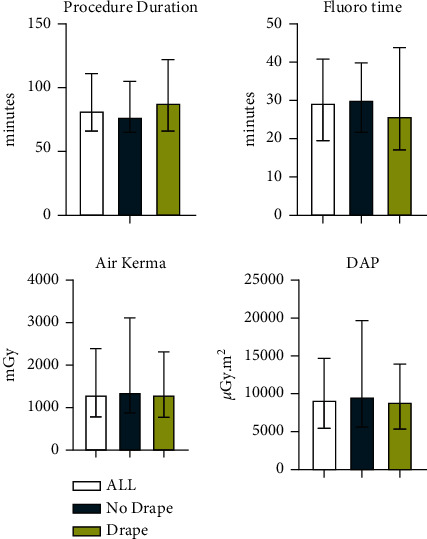
Median and interquartile ranges for procedure time (defined as the time from sheath-in to catheter-out), fluoroscopy time, air kerma, and dose area product (DAP) in all procedures and in the two groups. Bars represent median values with interquartile range.

**Figure 2:**
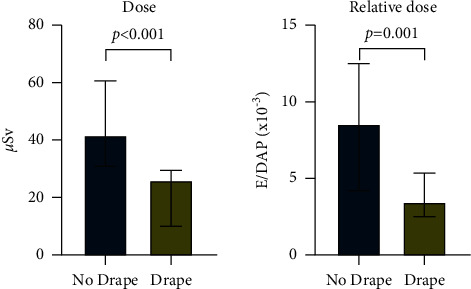
Median operator chest dose and relative operator chest dose normalized for dose area product (x10^−3^). Bars represent median values with interquartile range.

Sixty consecutive CTO procedures were included between October 2019 and November 2020. Baseline characteristics, CTO complexity (Table 1), fluoroscopy time, air kerma (*K*_a,r_, mGy), and DAP (*μ*Gy.m [2]) use were similar in the two groups (Figure 1). Fluoroscopy times were similar to previously published data [7, 8]. Dual access, with bilateral injections, was used in 88% of procedures. The use of MXPD was associated with 37% reduction in operator dose (weighted median dose 26.0 (IQR 10.00–29.47) *μ*Sv in the drape group versus 41.8 (IQR 30.82–60.59) *μ*Sv in the no drape group; *P* < 0.001) and a 60% reduction in relative operator dose (median dose 3.5 (IQR 2.5–5.4) E/DAP x 10^−3^ in the drape group versus 8.6 (IQR 4.2–12.5) E/DAP x 10^−3^ in the no drape group; *P*=0.001) (Figure 2). Finally, we performed a pooled patient-level analysis combining the current CTO PCI data with the previously published data from the parallel MXPD study in non-CTO PCI [4]. Using factorial ANOVA, we could show that MXPDs were equally effective at reducing operator radiation in both non-CTO and CTO procedures (*P* value for interaction 0.963).

## 4. Discussion

In this prospective randomized trial, we report that CTO operator radiation is reduced by 37% with the MAVIG X-ray protective drape (MXPD), and when DAP is taken into consideration, the effective dose for the CTO operator is reduced by 60%. These findings are in line with a previously published study with MXPD, which demonstrated a 57% reduction in relative operator radiation dose during routine cardiac catheterization and PCI [4].

Several studies have demonstrated that operator radiation can be significantly reduced when using a lead or lead-equivalent drape over the pelvis of the patient [2, 3, 9]. However, there are very few data regarding the effectiveness of these devices in reduction of radiation for CTO operators. Murphy et al. [7] demonstrated that the RADPAD (Worldwide Innovations and Technologies, Inc., Lenexa, KS) reduced operator radiation dose by 50% in a RCT including 40 CTO cases. Similarly, RADPAD shields significantly reduced operator radiation in a RCT of coronary procedures including 35 CTO PCIs [3], and Shorrock et al. [8] reported that RADPAD reduced CTO operator radiation dose to levels comparable to radiation dose during non-CTO procedures without a pelvic drape. Similarly, our data support the routine use of pelvic X-ray protective devices during CTO procedures to reduce operator radiation without increasing patient radiation (as measured by DAP).

The MXPD can be used under the sterile sheets of the patient or can be placed in a commercially available sterile plastic sleeve. This means that the MXPDs are reusable with a significant cost reduction in the long-term compared with RADPAD. The advantage of the sleeve system is that the position of the drape can easily be adjusted to ensure that it does not come into the field of view. However, in obese patients, it has a tendency of sliding out of position.

The study has some limitations, which have been discussed previously [4]. One additional limitation in this study is the small sample size. However, this remains the largest prospective study investigating operator radiation protection during CTO procedures. Furthermore, using a pooled patient-level analysis, we found that the drape was equally effective in this small study population as in the earlier large RCT [4].

## 5. Conclusions

This is the first CTO-dedicated randomized-controlled trial measuring operator radiation dose reduction with and without a pelvic radiation protection device. The pelvic MAVIG X-ray protective drapes reduced the effective CTO operator radiation dose by more than half.

## Figures and Tables

**Table 1:** Patient, theatre, and procedural details.

	All (*n* = 60)	No drape (*n* = 30)	Drape (*n* = 30)
Patient details			
Age (mean ± SD)	66 ± 11	68 ± 10	64 ± 12
Male	43 (72)	20 (67)	23 (77)
Weight (kg)	78.9 ± 15.5	80.6 ± 15.6	77.2 ± 15.8
Height (m)	1.7 ± 0.1	1.7 ± 0.1	1.7 ± 0.1
Body mass index (kg/m^2^)	24 ± 9.7	23.5 ± 11.6	24.3 ± 7.7
Diabetes	12 (20)	8 (27)	4 (13)
Hyperlipidemia	50 (83)	28 (93)	22 (73)
Hypertension	42 (70)	19 (63)	23 (77)
Prior PCI	29 (48)	16 (53)	13 (43)
Prior CABG	8 (13)	5 (17)	3 (10)

Procedure details			
Second operator took over^*∗*^	16 (27)	9 (30)	7 (23)
Radial Access	50 (83)	25 (83)	25 (83)
Femoral access	46 (77)	23 (77)	23 (77)
Bilateral injections	53 (88)	26 (87)	27 (90)
7F access	59 (98)	29 (97)	30 (100)

CTO details			
Left anterior descending artery	14 (23)	8 (27)	6 (20)
Right coronary artery	35 (58)	18 (60)	17 (57)
Circumflex artery	11 (18)	4 (13)	7 (23)
Complications	0 (0)	0 (0)	0 (0)
J-CTO score	2.1 ± 1.2	2.23 ± 1.3	1.9 ± 1.2

Values are number (%) or mean ± SD. SD, standard deviation; PCI, percutaneous coronary intervention; CABG, coronary artery bypass grafts. ^*∗*^Second operator took over a part of the procedure and the first operator moved to the second operator position.

## Data Availability

The data used to support the findings of this study were collected from the University Hospitals Leuven Cardiac Catheterization Laboratory and are made available from the corresponding author upon request.

## Abbreviations

CTO: Chronic total occlusion
DAP: Dose area product
Gy: Gray
J-CTO: Japan chronic total occlusion
*K*_a,r_: Air kerma
MXPD: MAVIG X-ray protective drapes
PCI: Percutaneous coronary intervention
RCT: Randomized-controlled trial.
